# Factor XI Deficiency Alters the Cytokine Response and Activation of Contact Proteases during Polymicrobial Sepsis in Mice

**DOI:** 10.1371/journal.pone.0152968

**Published:** 2016-04-05

**Authors:** Charles E. Bane, Ivan Ivanov, Anton Matafonov, Kelli L. Boyd, Qiufang Cheng, Edward R. Sherwood, Erik I. Tucker, Stephen T. Smiley, Owen J. T. McCarty, Andras Gruber, David Gailani

**Affiliations:** 1 Department of Pathology, Microbiology and Immunology, Vanderbilt University, Nashville, Tennessee, United States of America; 2 Department of Bioengineering and Organic Chemistry, Tomsk Polytechnic University, Tomsk, Russia; 3 Department of Anesthesiology, Vanderbilt University, Nashville, Tennessee, United States of America; 4 Aronora, Inc., Portland, Oregon, United States of America; 5 National Institute of Allergy and Infectious Disease, Bethesda, Maryland, United States of America; 6 Department of Biomedical Engineering, Oregon Health & Science University, Portland, Oregon, United States of America; University of Kentucky, UNITED STATES

## Abstract

Sepsis, a systemic inflammatory response to infection, is often accompanied by abnormalities of blood coagulation. Prior work with a mouse model of sepsis induced by cecal ligation and puncture (CLP) suggested that the protease factor XIa contributed to disseminated intravascular coagulation (DIC) and to the cytokine response during sepsis. We investigated the importance of factor XI to cytokine and coagulation responses during the first 24 hours after CLP. Compared to wild type littermates, factor XI-deficient (FXI^-/-^) mice had a survival advantage after CLP, with smaller increases in plasma levels of TNF-α and IL-10 and delayed IL-1β and IL-6 responses. Plasma levels of serum amyloid P, an acute phase protein, were increased in wild type mice 24 hours post-CLP, but not in FXI^-/-^ mice, supporting the impression of a reduced inflammatory response in the absence of factor XI. Surprisingly, there was little evidence of DIC in mice of either genotype. Plasma levels of the contact factors factor XII and prekallikrein were reduced in WT mice after CLP, consistent with induction of contact activation. However, factor XII and PK levels were not reduced in FXI^-/-^ animals, indicating factor XI deficiency blunted contact activation. Intravenous infusion of polyphosphate into WT mice also induced changes in factor XII, but had much less effect in FXI deficient mice. *In vitro* analysis revealed that factor XIa activates factor XII, and that this reaction is enhanced by polyanions such polyphosphate and nucleic acids. These data suggest that factor XI deficiency confers a survival advantage in the CLP sepsis model by altering the cytokine response to infection and blunting activation of the contact (kallikrein-kinin) system. The findings support the hypothesis that factor XI functions as a bidirectional interface between contact activation and thrombin generation, allowing the two processes to influence each other.

## Introduction

Inflammation and coagulation are key host defense processes that reinforce each other during the initial response to infection. Pattern-recognition molecules on host cells, such as toll-like receptors, interact with microorganism structures, initiating cytokine transcription [1]. The coagulation protease thrombin forms at injury sites, catalyzing fibrin formation that creates a barrier to microorganism spread [2]. Cytokine up-regulation of tissue factor on leukocytes and vascular endothelium enhances thrombin generation [3–7], and thrombin, in turn, cleaves protease-activated receptors (PARs) on a variety of cells, promoting expression of inflammatory mediators [3,5–8]. These processes are critical for an appropriate response to injury but can be destructive when not localized. Sepsis, a pathologic systemic response to infection, affects ~20 million people each year, with death occurring in 25% of severe cases [9–12]. The intense cytokine response and the thrombotic and hemorrhagic perturbations of coagulation that are features of sepsis are major contributors to tissue damage and mortality in this syndrome [1,5–8].

FXI (FXI) is the zymogen of the protease factor XIa (FXIa), a part of the blood plasma system that regulates thrombin generation [13–15]. FXI may be converted to FXIa by thrombin during blood coagulation in response to injury (hemostasis). Alternatively, FXI may be activated by the protease factor XIIa (FXIIa) during a process called contact activation [16–18]. Contact activation is not required for hemostasis but may contribute to inflammation and pathologic coagulation [14,19]. Previously, we reported that FXI deficiency improved survival during sepsis induced by cecal ligation and puncture (CLP) in mice [20]. There was evidence of disseminated intravascular coagulation (DIC) in wild type (WT) mice that was not present in FXI-deficient mice after CLP. An anti-FXI IgG (14E11) reduced DIC in FXI-deficient mice and, in addition, reduced plasma levels of interleukin-6 (IL-6) and tumor necrosis factor-α (TNF-α) measured 12 hours post-CLP, by ~50% [21]. It was not clear if FXI inhibition or an off-target effect of 14E11 was responsible for the reduced cytokine levels.

Inflammatory and hemodynamic changes occur within the first few hours after CLP in mice [22–24]. The purpose of the current study was to investigate the contribution of FXI to cytokine and coagulation responses during the early stages of CLP. While significant differences in cytokine responses between WT and FXI^-/-^ mice were noted as early as four hours post-CLP, there was surprisingly little evidence for a consumptive coagulopathy in animals of either genotype. Furthermore, while there was evidence of contact activation in the plasma of WT mice after CLP, the process appeared to be blunted in FXI-deficient mice. The results suggest that the beneficial effect of FXI deficiency in the sepsis model is related to an effect on the cytokine response and contact activation, and not necessarily to an antithrombotic effect.

## Materials and Methods

### Ethics statement on use of mice

The Institutional Animal Care and Use Committee at Vanderbilt University approved all experiments with mice (protocols M/10/290 and M/10/298). Surgery was performed under isoflurane (CLP) or sodium pentobarbital (polyphosphate [poly-P] infusion) anesthesia, and all efforts were made to minimize suffering. Animals were housed under a 12-hour day/night cycle with unrestricted access to food and water.

### Cecal Ligation and Puncture

For CLP survival and time course studies [20,21,25], C57Bl/6 mice heterozygous for a null FXI allele (FXI^+/-^) were crossed to generate wild type (WT, FXI^+/+^), heterozygous null (FXI^+/-^), and homozygous null (FXI^-/-^) mice [26]. Age-matched (3 to 6 months) male littermates were used. Mice were anesthetized with isoflurane. The cecum was exteriorized through a midline celiotomy, ligated with 4–0 coated Vicryl^™^ either 1 cm from the distal end (low-grade injury) or at the base distal to the ileocecal valve (high-grade injury), then punctured twice with a sterile 21-gauge needle. After a drop of cecal contents was expressed, the organ was returned to the abdominal cavity, the abdominal wall was closed with 4–0 Vicryl^™^, and skin was closed with wound clips. Mice received 1 mL warm 0.9% NaCl subcutaneously (SC) post-surgery. After consultation with a veterinarian, it was decided not to use analgesics because they alter the process being studied. After CLP mice were evaluated every 6 hours, and received 1 mL warm 0.9% NaCl SC daily. At each inspection, animals that (1) were moribund (no response to stimulation), (2) in respiratory distress, (3) were cool to touch, (4) had dusky discoloration of the tail or ears, or (5) had lost >30% body weight were euthanized (CO2 inhalation). Less than 5% of mice met these criteria. For time course experiments, mice underwent high-grade CLP or sham (cecum manipulated without ligation or puncture) surgery. At specific times post-CLP mice were anesthetized with isoflurane, blood was collected by cardiac puncture, and the animals were sacrificed by cervical dislocation while under anesthesia. No animals met criteria for euthanasia prior to reaching designated time points.

### Measurements of Blood Parameters

Blood was drawn into a 1/10th volume of 100 mM EDTA. Cell counts were measured on a Hemavet^®^ 950 FS analyzer (Drew Scientific). Plasma cytokines (IL-1β, IL-6, IL-10, TNFα) were measured on a Luminex 100 system (Luminex Corporation, Austin, TX). Mouse specific ELISAs were used to measure plasma levels of serum amyloid P (SAP), thrombin-antithrombin (TAT) complex and fibrinogen (all from Innovative Research, Novi, MI).

Changes in plasma levels of FXI, factor XII (FXII) and prekallikrein (PK) were determined by chemiluminescent western blots. Plasmas (1 μL) were size fractionated on 10% polyacrylamide-SDS gels, and transferred to nitrocellulose. FXI was detected with a biotinylated anti-mouse-FXI IgG (14E11) [27] and streptavidin-HRP. PK was detected with HRP-conjugated sheep-anti-human PK and FXII with HRP-conjugated goat-anti-human FXII IgG (Affinity Biologicals, Ancaster, ON). Blots were analyzed with BioRad Quantity 1 software. Mean signals for baseline samples were assigned values of 100%, and served as references for signals at other time points.

### Histology

Liver, spleen, thymus, kidney, and brain were collected from controls and from mice sacrificed 4, 8, or 24 hrs post-CLP or sham surgery, fixed in 10% buffered neutral formalin, embedded in paraffin, sectioned, and stained with hematoxylin-eosin. Slides were submitted to a veterinary pathologist (KLB) with numeric codes to facilitate blinding. Twenty high-power fields were evaluated for each sample for fibrin accumulation, platelet clumping, inflammation, lymphoid apoptosis, hemorrhage, and necrosis.

### Chromogenic assay for FXII activation

Human FXII, FXIa, α-kallikrein and high molecule weight kininogen (HK) were from Enzyme Research Laboratory (South Bend, IN). Genomic DNA was isolated from human leukocytes, and total RNA from mouse liver. Poly-P (60–100 phosphate units) was a gift from Thomas Renné (Karolinska Institute). Activation of FXII (200 nM) was studied in 20 mM HEPES, pH 7.4, 100 mM NaCl, 0.1% PEG-8000, ZnCl2 (10 μM) at 37°C. Reactions included DNA (5.0 μg/ml), RNA (5.0 μg/ml), poly-P (20 μg/ml), HK (20 nM), α-kallikrein (2.0 nM), or FXIa (1.0 nM). At various times, 20 μl of reactions were mixed with 10 μl Polybrene (0.4 mg/ml) and 40 nM of an anti-kallikrein IgG H03 [28] (for reactions with α-kallikrein) or 40 μM aprotinin (for reactions with FXIa). Ten microliters S-2302 (2 mM in H2O) was added and ΔOD 405 nm was monitored on a microplate reader. Results were compared to a standard curve prepared with pure FXIIa.

### Poly-P-induced FXII activation in mice

C57Bl/6 WT mice, and mice lacking FXII [29], PK [30], or FXI [26] were anesthetized with sodium pentobarbital. The abdomen was opened, and poly-P (60 mg/kg) in 100 μL of PBS was infused into the inferior vena cava over 30 seconds. Five minutes later, blood was collected into 1/10^th^ volumes of 3.2% sodium citrate, and plasma was prepared by centrifugation. Mice were sacrificed by cervical dislocation after phlebotomy while under anesthesia. Plasma samples (1 μL) were size fractionated under non-reducing conditions on 12% polyacrylamide-SDS-gels, transferred to nitrocellulose paper, and developed with HRP-conjugated goat anti-human FXII IgG (Affinity Biologicals). Detection was by chemiluminescence.

### Statistical Analyses

Data were analyzed using GraphPad Prism^®^ 5.0 software. Survival differences were evaluated by log-rank test. Differences in continuous variables (cytokines, plasma proteins, platelet counts) were assessed with a non-parametric Mann-Whitney test. For densitometry of Western blots, the one-sample t-test was used to differentiate between mean intensity for each group from signal intensity at the pre-surgical baseline (assigned a value of 100%). Data are presented as mean ± standard error of the mean (SEM). A *p* value of < 0.05 was considered significant for all analyses.

## Results

### FXI deficiency affects early cytokine responses to CLP

**Fig 1:**
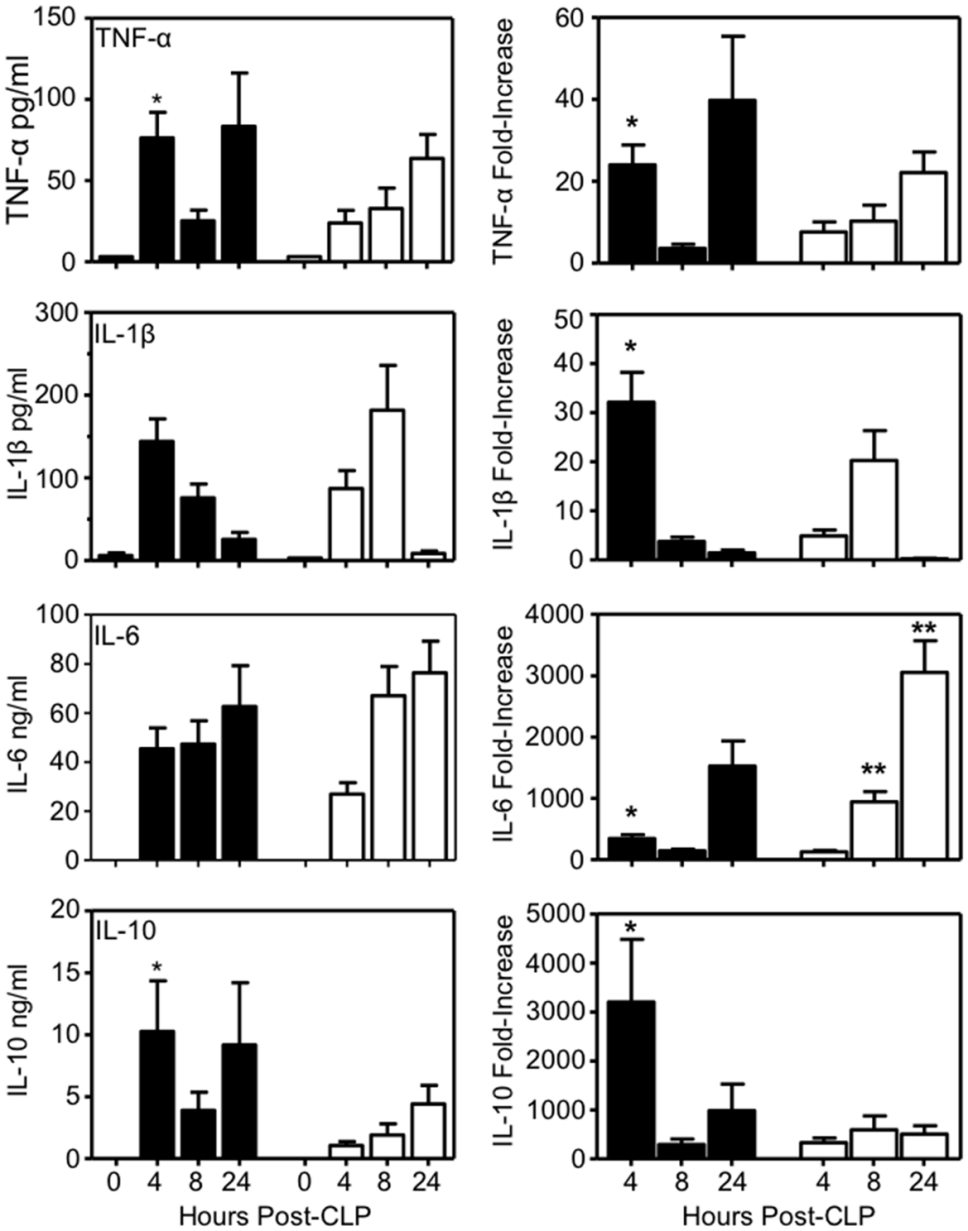
Plasma cytokine levels after CLP. Shown are plasma concentrations of TNFα, IL-1β, IL-6 and IL-10 in WT (black bars) and FXI^-/-^ (white bars) littermates at various times after high-grade CLP. Absolute cytokine levels are shown in the left-hand column and fold-increases in cytokines compared to sham treatment are shown in the right-hand column. Eight or nine mice were tested at each time point for each genotype for CLP, and three were used at each time point for sham surgery. Plasma TNFα **(***p* = 0.006) and IL-10 **(***p* = 0.0003) levels were significantly greater in WT mice than in FXI^-/-^ mice 4 hr post-CLP. Fold-increases in plasma levels of TNFα, **(***p* = 0.009), IL-1β, **(***p* = 0.009), IL-6 (*p* = 0.003) and IL-10 (*p* = 0.0003) were significantly greater in WT mice than in FXI^-/-^ mice 4 hr post-CLP. For IL-6, FXI^-/-^ mice had significantly greater fold-increases in plasma levels 8 (**p = 0.005) and 24 hr (**p = 0.04) post-CLP. Error bars represent SEM.

A pilot study was performed to identify cytokines with the largest increases after CLP in WT mice, and to determine optimal time points for measurement. TNF-α, IL-1β, and IL-6, and IL-10 displayed the greatest increases (S1 Fig), and were chosen for studies of WT and FXI^-/-^ littermates. Absolute cytokine levels are shown in the left hand column of Fig 1, while the right-hand column presents cytokine data as fold-increase compared to sham surgery to illustrate the impact of CLP over and above anesthesia and celiotomy alone. Plasma TNF-α and IL-10 levels were significantly greater in WT mice than in FXI^-/-^ mice 4 hours post-CLP (*p* = 0.006 and 0.0003, respectively), and there was a trend toward higher IL-1β and IL-6 levels four hours post-CLP in WT animals (*p* = 0.16 and 0.06, respectively). Later increases out to 24 hours for TNF-α, IL-6, and IL-10 were comparable between genotypes. Peak IL-1β levels were similar between WT and FXI^-/-^ mice, however, they occurred later (8 hrs) in FXI^-/-^ mice than in WT mice (4 hrs). Fold-increases in cytokine levels in CLP-treated WT mice were significantly greater than in FXI^-/-^ mice four hours post-CLP for all four cytokines (TNF-α *p* = 0.009, IL-1β *p* = 0.0009, IL-6 *p* = 0.003, and IL-10 *p* = 0.0003). For IL-6, the fold-increase at 8 and 24 hours post-CLP were greater in FXI^-/-^ mice than WT mice (*p* = 0.04).

**Fig 2:**
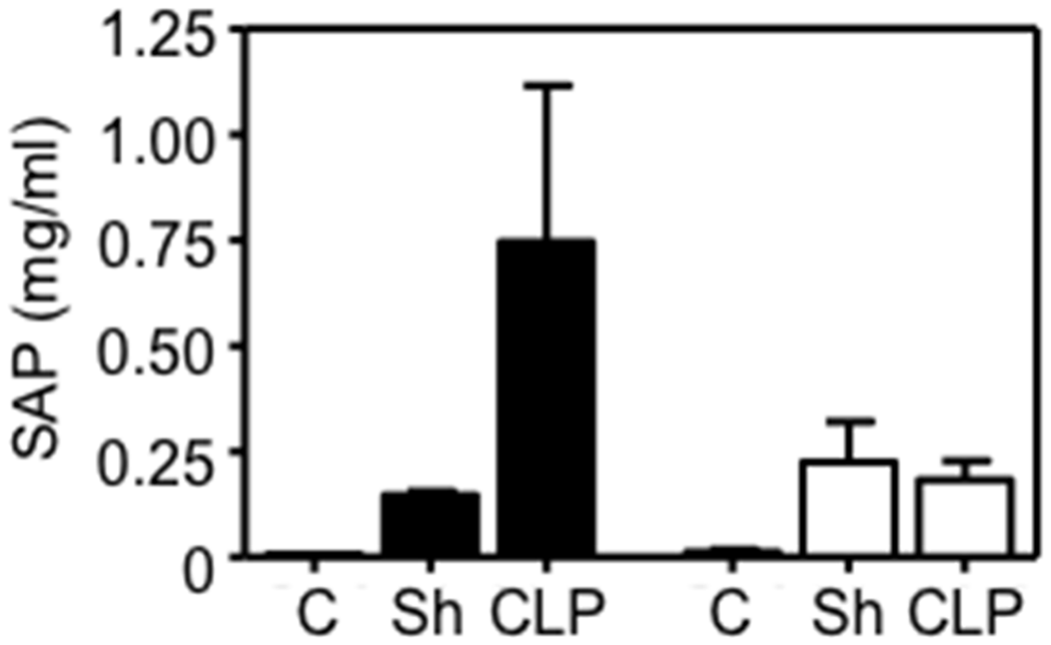
Serum amyloid P levels post-CLP. Plasma levels of SAP measured by ELISA in control mice (C, n = 4) not undergoing surgery, and 24 hr post-CLP (n = 8–9) or sham (Sh n = 3) surgery. Black bars are results for WT mice and white bars for FXI^-/-^ mice. Error bars represent SEM.

SAP is a homolog of C-reactive protein (CRP), a member of the pentraxin family of proteins used to evaluate inflammatory disorders in humans [31]. Like CRP, SAP is an acute phase protein. In WT mice, SAP levels 24 hours post-CLP were 5- to 6-fold greater than in mice undergoing sham surgery (Fig 2). SAP concentrations in FXI^-/-^ mice 24 hours post-CLP were comparable to sham-treated mice (Fig 2), consistent with the impression that the altered cytokine response in FXI^-/-^ mice is associated with reduced inflammation when compared with WT mice.

### FXI deficiency confers a survival advantage after CLP

**Fig 3:**
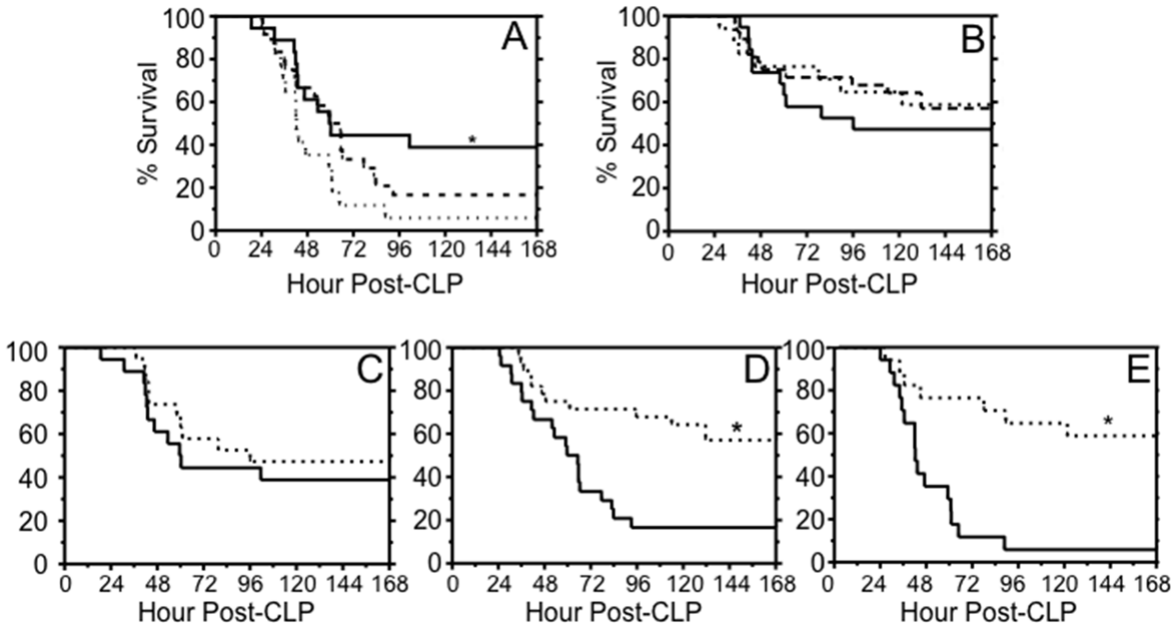
Survival after CLP. **(A)** Survival of male WT (…, n = 17), FXI^+/-^ (---, n = 24), or FXI^-/-^ (—, n = 18) mice after high-grade CLP. *p* = 0.03 (*) for WT vs. FXI^-/-^. **(B)** Survival for male WT (n = 17), FXI^+/-^ (n = 28), or FXI^-/-^ (n = 19) mice after low-grade CLP (*p* = 0.4 for WT vs. FXI^-/-^, *p* = 1.0 for WT vs. FXI^+/-^, *p* = 0.5 for FXI^-/-^ vs. FXI^+/-^). **(C-E)** Survival for male mice after high-grade (—) or low-grade (…) injury. **(C)** FXI^-/-^, **(D)** FXI^+/-^ and **(E)** WT mice. There was no difference in survival for FXI^-/-^ mice after low- or high- grade injury (*p* = 0.47). Survival was significantly different between the two levels of injury for FXI^+/-^ (*p* = 0.001) and WT (*p* = 0.0002) mice. Curves were compared by log-rank test.

Survival studies were performed at two levels of injury (high- and low-grade), using FXI^-/-^, FXI^+/-^, and WT littermates to address a concern that earlier observations of a survival difference between unrelated FXI^-/-^ and WT mice [20] may have been due to differences in gut flora or background genetics. Survival at seven days post-high grade CLP was 6% in WT mice, 17% in FXI^+/-^ mice, and 39% in FXI^-/-^ mice (Fig 3A). FXI^-/-^ mice had significantly better survival than WT mice (*p* = 0.03), while differences in survival in FXI^-/-^ and FXI^+/-^ mice were not significantly different (*p* = 0.09). Interestingly, survival was similar for all genotypes after low-grade CLP (Fig 3B, *p* = 0.3 for WT vs. FXI^-/-^, *p* = 1.0 for WT vs. FXI^+/-^). Indeed, survival was unrelated to injury grade in FXI^-/-^ mice (Fig 3C *p* = 0.47), while injury grade influenced survival for FXI^+/-^ (Fig 3C, *p* = 0.001 for FXI^+/-^) and WT (Fig 3D, *p* = 0.0002 for WT) mice. These data suggest that FXI contributes to mortality during more severe sepsis in this model.

### Evaluation for CLP-induced coagulopathy

**Fig 4:**
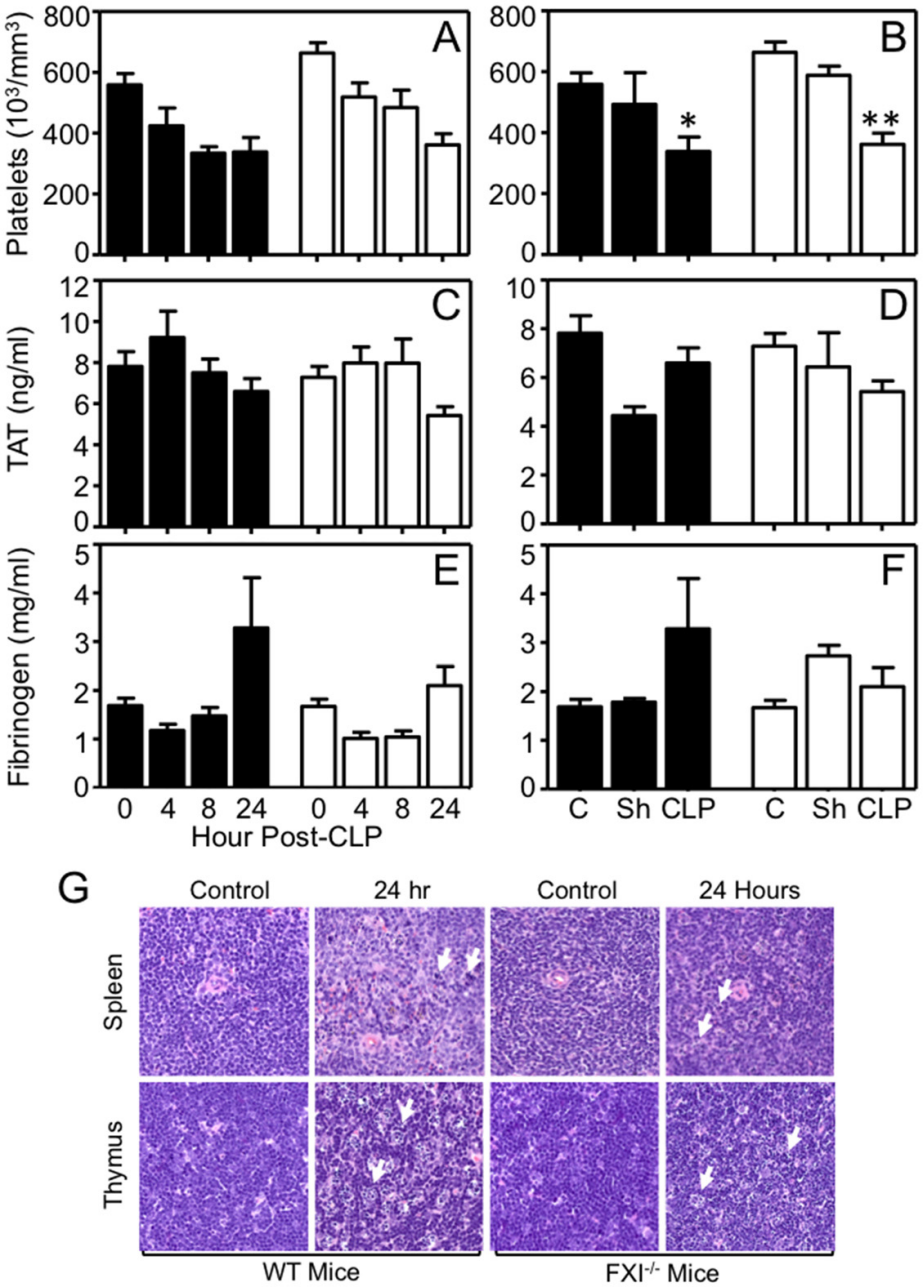
Effects of CLP on markers of coagulation. **(A,B)** Whole blood platelet counts, **(C,D)** plasma thrombin-antithrombin complex (TAT) levels, and **(E,F)** plasma fibrinogen levels in WT (black bars) and FXI^-/-^ (white bars) mice after CLP or sham surgery. Panels A, C and E show values at various times post-CLP. Panels B, D and F compare values 24 hour post-CLP or sham (Sh) surgery to 0 hr controls. CLP induced significant platelet reductions in WT (*p = 0.003) and FXI^-/-^ (**p = 0.001) mice. For panels **A** to **F**, error bars represent SEM. **(G)** Photomicrographs (400X magnification) of paraffin embedded sections of spleen and thymus stained with hematoxylin and eosin before, and 24 hours after, CLP in WT and FXI^-/-^ mice. Apoptotic changes (indicated by white arrows) were present in both organs for mice of both genotypes 24 hrs post-CLP.

Platelet counts were reduced 40–45% compared to baseline 24 hours post-CLP in WT and FXI^-/-^ mice (Fig 4A, WT baseline 559 ± 37 X 10^3^/μL, 24 hrs post-CLP 339 ± 47 X 10^3^/μL, *p* = 0.003; FXI^-/-^ baseline 664 ± 34 X 10^3^/μL, 24 hrs post-CLP 362 ± 37 X 10^3^/μL, *p* = 0.001). Platelet count changes were significantly greater than with sham surgery (Fig 4B). Plasma TAT levels did not increase significantly above baseline for either genotype (Fig 4C), and were not different from sham-treated animals (Fig 4D). Fibrinogen was modestly increased above baseline 24 hours post-CLP (Fig 4E and 4F, WT: baseline 1.7 ± 0.2 mg/mL, 24 hrs 3.3 ± 1.0 mg/mL; FXI^-/-^: baseline 1.7 ± 0.2, 24 hrs 2.1 ± 0.4), with a trend toward higher levels in WT mice. Fibrinogen levels rise as an acute phase response, and the difference could reflect a greater inflammatory response in WT mice.

At necropsy 24 hrs post-CLP, evidence of lymphocyte apoptosis, a common finding in murine sepsis [32], was noted in spleen and thymus for both genotypes (Fig 4G). There was no evidence of hemorrhage. Histologic analysis of liver, kidney, brain, spleen, and thymus did not reveal microvascular thrombus accumulation (S2 Fig), which is considered a diagnostic feature of DIC in laboratory animals [33]. These data show that in this study consumptive coagulopathy was not a prominent feature of sepsis during the first 24 hours after high-grade CLP injury.

### Effects of CLP on contact factors

When blood is exposed to anionic “surfaces”, FXII is converted to the protease FXIIa by a process called contact activation [16–18]. During sepsis, blood may be exposed to a variety of polyanions that induce contact activation, including poly-P from platelets or microorganisms [34,35], and extracellular DNA and RNA from damaged tissue and chromatin extruded from activated neutrophils (neutrophil extracellular traps) [36,37]. FXIIa converts the homologous proteins prekallikrein (PK) and FXI, to α-kallikrein and FXIa, respectively. In classic contact activation, α-kallikrein amplifies the process by activating FXII, and contributes to inflammation and increased vascular permeability by cleaving high molecular weight kininogen (HK) to liberate bradykinin. FXIa, in contrast, contributes to thrombin generation through factor IX activation, and is not thought to have a significant role in FXII activation.

**Fig 5:**
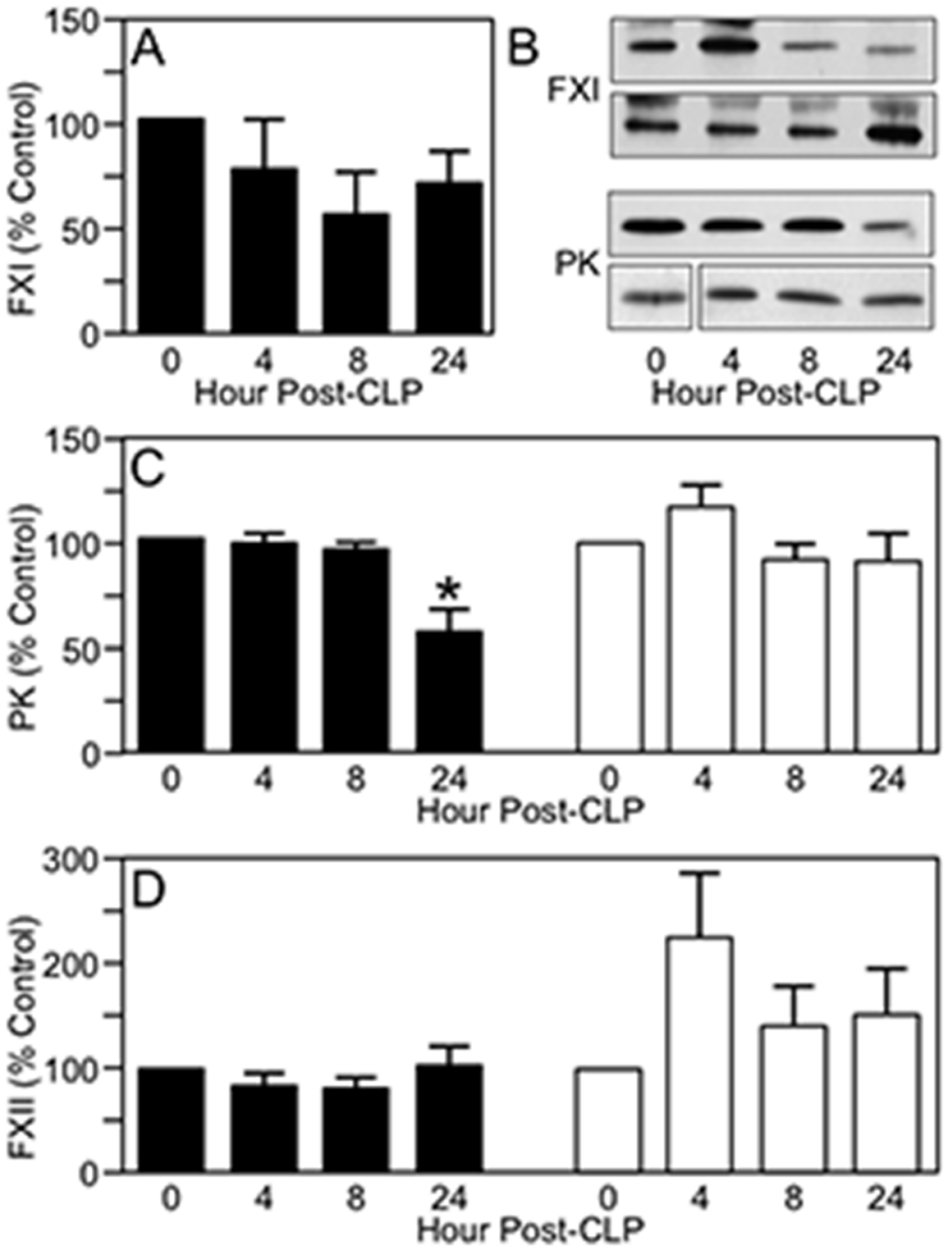
The effect of CLP on plasma contact proteases. Plasma FXI, PK and FXII levels at various times after high-grade CLP were determined using densitometry of western blots and are reported as percent of baseline (0 hr) control. **(A)** Plasma FXI levels after CLP in WT mice **(B)** Examples of western blots for FXI and PK for WT mouse plasma at various times after CLP (upper panels of each pair) or sham surgery (lower panels of each pair). **(C)** Plasma PK levels after high-grade CLP in WT (black bars) or FXI^-/-^ (white bars) mice. The asterisk above the 24 hr bar for PK in WT animals indicates the value is significantly different than 0 hr control (*p*<0.05). **(D)** Plasma FXII levels after high-grade CLP in WT (black bars) or FXI^-/-^ (white bars) mice. In panels A, C, and D, each bar represents data for eight mice. For all panels, error bars represent SEM.

In humans and laboratory animals, endotoxin-induced activation of the contact proteases (FXII, PK and FXI) is associated with reduction in their plasma antigen levels [38–40]. In WT mice, FXI decreased by ~50% by 8 hours after CLP (Fig 5A and 5B), and PK was comparably reduced by 24 hours (Fig 5B and 5C). While FXII levels did not change appreciably from baseline in WT mice post-CLP, the levels increased modestly in FXI^-/-^ mice and in sham-treated animals (not shown) suggesting a reaction to surgery. The data indicate there is activation and consumption of FXII in WT mice after CLP that was not as prominent in FXI^-/-^ mice. Interestingly, PK levels were not reduced 24 hours post-CLP in FXI^-/-^ mice. Taken as a whole, the data are consistent with activation of the contact proteases during CLP-induced sepsis in WT mice, but indicate that FXI deficiency blunted this process. A possible mechanism that would explain these findings is that FXIa, like its homolog α-kallikrein, supports FXII activation.

### FXII activation by α-kallikrein and FXIa

**Fig 6:**
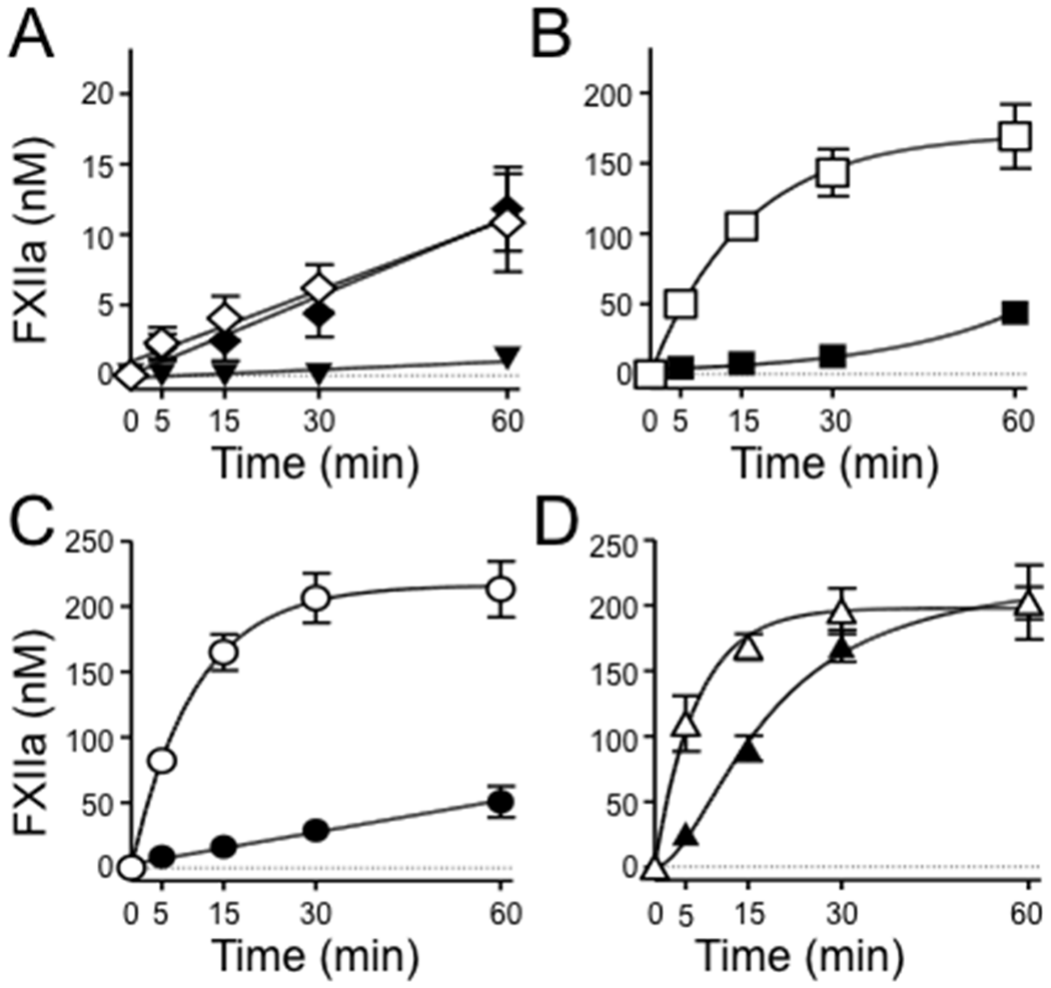
FXII activation by α-kallikrein or FXIa in the presence of polyanions. **(A)** FXII (200 nM) was incubated with vehicle (▼), 1nM FXIa (◇), or 2 nM α-kallikrein (◆). **(B-D)** FXII (200 nM) incubated with **(B)** 5 ug/ml DNA (□,➄), **(C)** 5 ug/ml RNA (○,➂) or **(D)** 20 μg/ml Poly-P (△,▲), in the presence of 1 nM FXIa (□,○,△) or 2 nM α-kallikrein (➄,➂,▲). At the indicated times, samples were tested for FXIIa activity by chromogenic assay. Error bars are +/− one standard deviation.

FXII activation by α-kallikrein or FXIa was tested in the presence or absence of the polyanions DNA, RNA, and poly-P. FXII was converted to FXIIa at comparable rates by α-kallikrein and FXIa in the absence of a polyanion (Fig 6A). The FXIa-mediated reaction was enhanced 56-fold by DNA (Fig 6B) and 90-fold by RNA (Fig 6C). With α-kallikrein, DNA and RNA enhanced FXII activation modestly (2.8 and 5.6-fold, respectively). In reactions with poly-P, α-kallikrein and FXIa contributions to FXII activation appeared similar, but were difficult to quantify because FXII autoactivation was prominent (Fig 6D). Because of the homology between FXI and PK [13], they can be difficult to separate chromatographically when purified from plasma. To address the concern that FXIa was contaminated with α-kallikrein, we ran reactions in the presence of an anti-kallikrein IgG (H03) that binds to the protease active site (S3A and S3B Fig) [28]. H03 blocked FXII activation by α-kallikrein (S3C Fig) but did not affect FXII activation by FXIa (S3D Fig).

**Fig 7:**
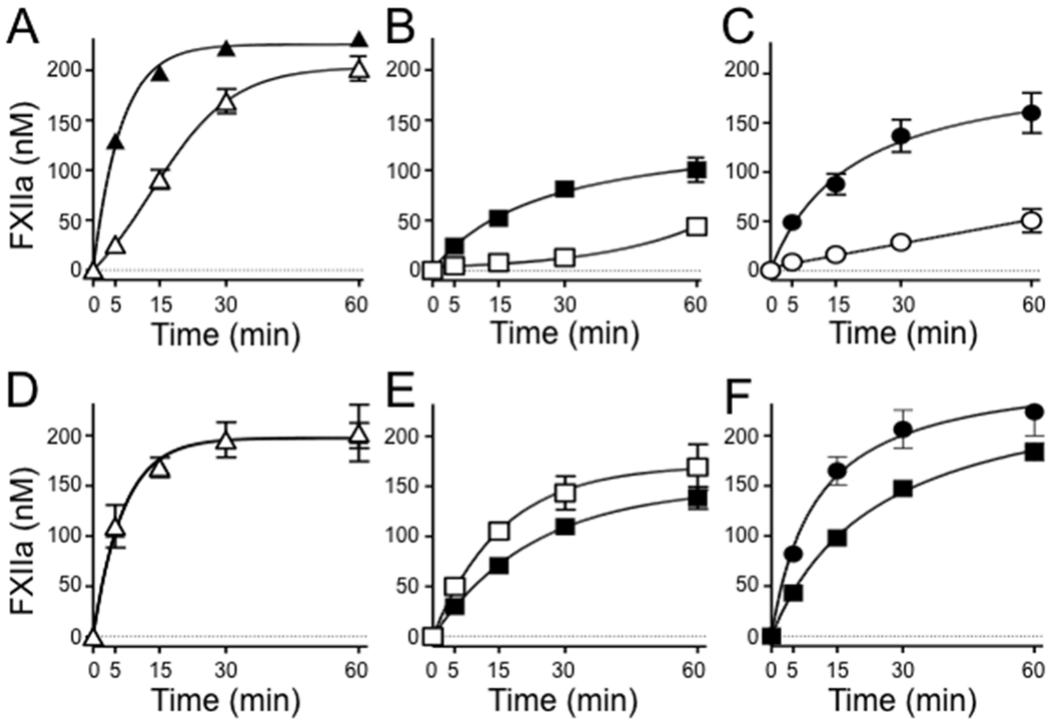
Effect of HK on FXII activation in the presence of polyanions. Plasma FXII (200 nM) was incubated with (**A-C**) 2 nM α-kallikrein or (**D-F**) 1 nM FXIa and **(A,D)** 20 μg/ml poly-P, **(B,E)** 5 μg/ml DNA or **(C,F)** 5 μg/ml RNA in the absence (○,□,△) or presence (➂,➄,▲) of 20 nM HK. At the indicated times, samples were tested for FXIIa activity by chromogenic assay. Error bars are +/− one standard deviation.

Most PK and nearly all FXI in plasma circulates as a complex with the glycoprotein HK [41–43]. HK is thought to be required for proper binding of PK and FXI to the contact surface during contact activation [44,45]. Consistent with this, HK enhanced FXII activation by α-kallikrein with Poly-P (Fig 7A), DNA (Fig 7B) or RNA (Fig 7C). However, HK had no appreciable effect on FXII activation by FXIa in the presence of Poly-P (Fig 7D), and a modest inhibitory effect with DNA (Fig 7E) or RNA (Fig 7F). These data suggest that α-kallikrein (in an HK-dependent manner) and FXIa (in an HK-independent manner) can both activate FXII *in vitro*, supporting the notion that FXIa may be an activator of FXII *in vivo*.

### Effects of poly-P infusion on FXII in mice

**Fig 8:**
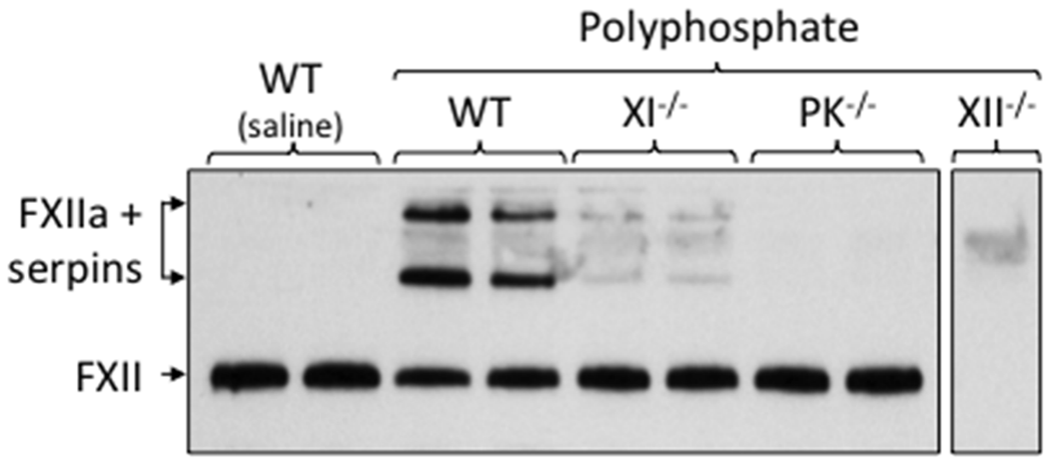
Poly-P-induced changes in FXII in mice. WT C57Bl/6 mice or C57Bl/6 mice lacking FXI (XI^-/-^), PK (PK^-/-^) or FXII (XII^-/-^) received a bolus infusion of phosphate buffered saline (saline) or PBS containing 60 μg of poly-P into the inferior vena cava. Five minutes later blood was drawn from the inferior vena cava into sodium citrate anticoagulant. Plasma samples were analyzed by western blot for FXII under non-reducing conditions. The blot contains samples for two mice of each genotype. The position of the FXII zymogen band is indicated on the left (FXII). Free FXIIa would also run in this position. The higher molecular mass FXII specific-species likely represent FXIIa in SDS-stable complexes with plasma serine protease inhibitors (serpins).

Intravenous infusion of poly-P induces rapid FXII-dependent thrombosis in mice [35]. Poly-P infusion into WT mice caused a modest decrease in the intensity of the band representing FXII on western blot, and produced higher molecular weight species that likely represent FXIIa in SDS-stable complexes with serpins such as C1-inhibitor and antithrombin (Fig 8). There were no bands on blots with plasma from poly-P treated FXII-deficient mice, supporting the premise that the high molecular mass bands in WT mouse plasma are specific FXII/XIIa signals. No changes were apparent in FXII in PK-deficient mice after poly-P infusion, consistent with the impression that PK is required for normal FXII turnover in healthy mice [46]. Surprisingly, FXI deficiency also affected poly-P-induced changes in FXII, although perhaps not quite as profoundly as did PK deficiency. Thus, both α-kallikrein and FXIa appear to be required for optimal FXII activation after poly-P infusion in mice, consistent with the observation that FXI deficiency led to reduced activation of contact proteases in the CLP model.

## Discussion

FXIa, despite its modest role in hemostasis, is implicated in pathologic coagulation in humans [19,47–50]. We previously observed evidence for disseminated intravascular coagulation (DIC) after CLP in WT mice, but not FXI^-/-^ mice or WT mice given the anti-FXI IgG 14E11 [20,21]. Our initial conclusion was that the survival advantage conferred by FXI deficiency was largely due to blunting of sepsis-induced DIC. However, WT mice treated with 14E11 also had lower plasma levels of TNFα and IL-6 after CLP than vehicle-treated mice [21]. The current study explored the importance of FXI in early cytokine and coagulation responses after CLP. A feature distinguishing it from earlier work is that WT and FXI-deficient littermates were used to reduce the likelihood that differences in gut flora influenced results. The main findings of the study are that (1) FXI deficiency has a major effect on the early cytokine response in sepsis, (2) DIC is not always a prominent feature of CLP-induced sepsis, and (3) FXI deficiency is associated with reduced FXII and PK activation after CLP. The data suggest that the survival benefit conferred by FXI-deficiency is more likely to be related to changes in cytokine responses and activation of the contact (kallikrein-kinin) system than to a reduction in consumptive coagulation.

In the present study the marked elevations in cytokines noted within four hours of CLP in WT mice were less pronounced in FXI^-/-^ mice. Furthermore, FXI deficiency altered the pattern and timing of cytokine release. Plasma TNFα and IL-10 expression in WT mice followed a biphasic pattern (Fig 1), with peaks at 4 and 24 hours post-CLP separated by lower levels at 8 hours. In FXI^-/-^ mice the responses for these cytokines followed a different trajectory, with a gradual rise over 24 hours and loss of the initial peak. WT and FXI^-/-^ mice displayed roughly similar patterns for plasma IL-1β and IL-6 levels, but the responses were delayed in FXI^-/-^ mice. The difference in levels of the acute phase protein SAP in WT and FXI^-/-^ mice 24 hours post-CLP supports the conclusion that the altered cytokine response in FXI^-/-^ mice was associated with less robust inflammation. But FXI deficiency may also have partially mitigated the immune dysfunction that accompanies sepsis. Ayala *et al* showed that macrophages collected from mice 24 hours post-CLP have reduced capacity to secrete IL-6 [51]. The observation that IL-6 levels were actually somewhat higher in FXI^-/-^ mice than WT mice 24 hours post-CLP may indicate that sepsis-induced immune dysfunction was greater in WT mice than in FXI^-/-^ mice.

In attempting to determine how FXIa contributed to cytokine production in CLP-induced sepsis, we focused on its known roles in thrombin generation and contact activation. Surprisingly, we did not observe histologic or biochemical evidence for DIC during the 24 hours post-CLP in WT or FXI^-/-^ mice, and specifically did not see increases in thrombin-antithrombin complex. Several factors may have contributed to these results, which are discrepant with our prior work. First, CLP does not consistently induce DIC in mice [23,52]. It is also possible that differences in gut flora between inbred lines were a factor in our original studies. Furthermore, our earlier work used a lower grade injury than the present study. These issues aside, the important point to take from the cumulative experience is that FXI-deficiency confers a survival advantage after CLP, regardless of the presence of DIC. This in turn suggests that the detrimental effect of FXI in this model is unrelated to consumptive coagulation. Corral *et al*. noted evidence for DIC in mice after CLP or lipopolysaccharide infusion [53]. Inhibition of the nuclear enzyme PARP-1 reduced inflammation and improved survival in both settings, but had little impact on DIC. They proposed that activation of coagulation can be independent of the inflammatory response, and that the coagulopathy had a minor influence on survival. Our work supports their conclusions.

**Fig 9:**
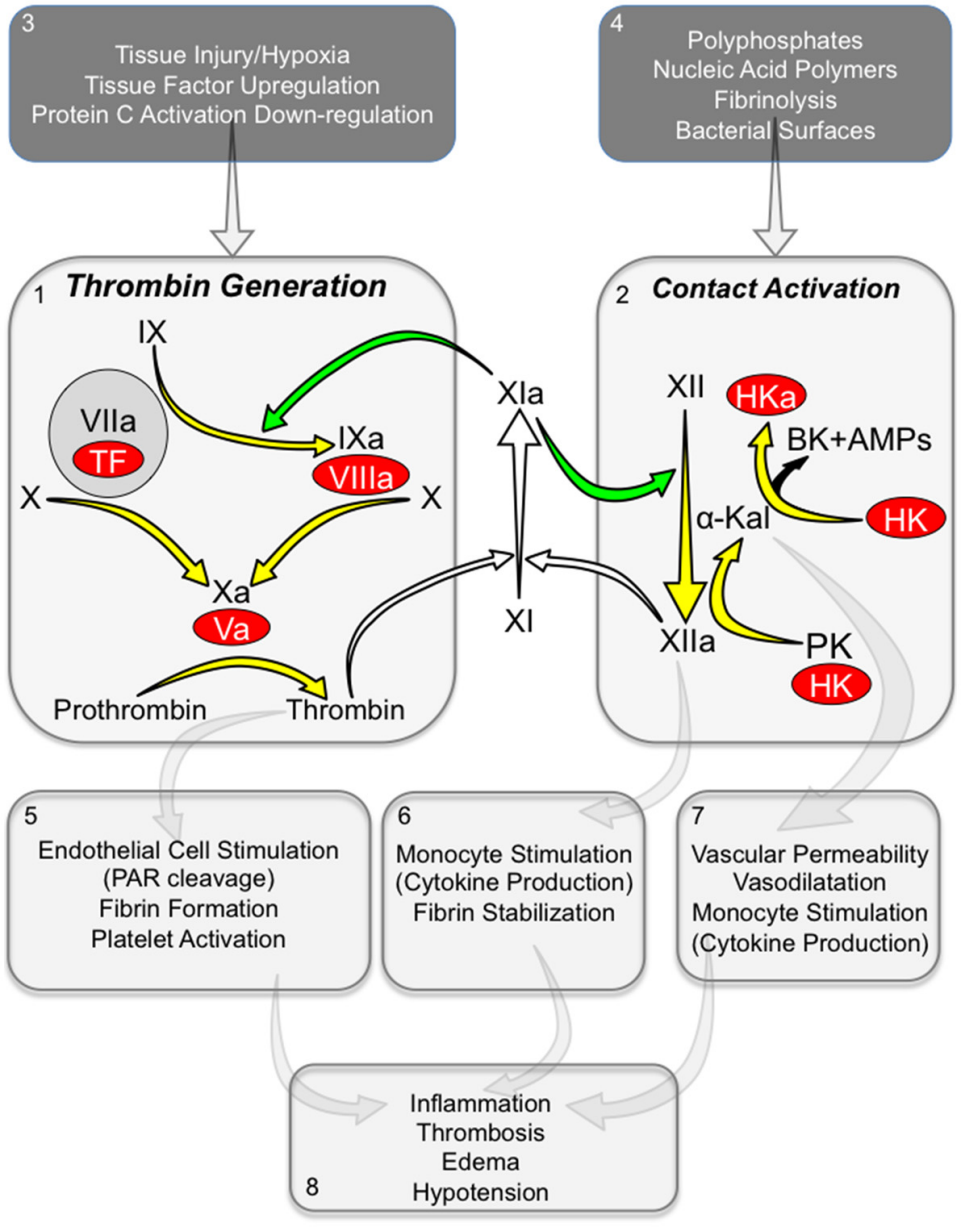
FXI in thrombin generation and contact activation during sepsis. *Thrombin Generation (Panel 1)*. Depicted are proteolytic reactions that generate thrombin at a site of vascular injury. The process is initiated by activation of factors X and IX by the factor VIIa/tissue factor (TF) complex. Vitamin-K dependent protease zymogens are shown in black type with the active protease forms indicated by a lower case “a”. The red ovals represent cofactors. During hemostasis, FXI is converted to FXIa by thrombin (white arrows). FXIa then activates FIX (green arrow). *Contact activation (Panel 2)*. On a surface, FXII and prekallikrein (PK) undergo reciprocal activation to FXIIa and α-kallikrein. High-molecular-weight kininogen (HK) serves as a cofactor for this reaction. FXIIa can promote thrombin generation by activating FXI (white arrow). α-kallikrein cleaves HK liberating bradykinin (BK) and antimicrobial peptides (AMPs). Data presented in this manuscript raise the possibility that FXIa can also contribute to contact activation through activation of FXII (green arrow). Panels 1 and 2 list factors that could trigger or enhance thrombin generation (1) or contact activation (2). Panels 5, 6, and 7 list processes mediated by thrombin (5), FXIIa (6) or α-kallikrein (7) that could contribute to the sepsis syndrome. Panel 8 lists some of the consequences of those processes.

Hypothetically, FXI could influence the course of sepsis in the absence of frank DIC by several mechanisms, some of which are diagramed in Fig 9. First, FXI is a component of the mechanism that forms thrombin during hemostasis (Fig 9, *panel 1*), and it is reasonable to assume that it contributes to thrombin generation during sepsis, even in the absence of clinical DIC. Thrombin upregulates cytokine production through cleavage of PARs on vascular and hematopoietic cells, and enhances inflammation through fibrin generation and platelet activation (Fig 9, *panel 5*). During tissue factor-initiated coagulation, FXI is converted to FXIa by thrombin. FXIa, in turn, sustains thrombin generation by activating factor IX. This process could be accentuated in sepsis through up-regulation of tissue factor expression, and through increased vascular permeability giving pro-coagulant proteins in blood easier access to cells that constitutively express tissue factor (Fig 9, *panel 3*) [3,4].

The reduced plasma levels of FXI, PK and FXII after CLP in WT mice are consistent with induction of contact activation (Fig 9, *panel 2*) [38–40]. FXIa could serve as a bridge that permits FXIIa to contribute to thrombin generation. During sepsis, a variety of promoters of FXII activation (Fig 9, *panel 4*), including poly-P [34] and nucleic acids from microorganisms and damaged tissues [36,37], plasmin generated during hyperfibrinolytic responses [54,55], and bacterial surfaces [56,57] could induce contact activation leading to FXI activation and, ultimately, to increased thrombin production. However, contact activation likely contributes to inflammation during sepsis through thrombin-independent mechanisms. Products of contact activation such as FXIIa and cleaved HK stimulate cytokine production in monocytes [58,59], while α-kallikrein cleaves HK liberating bradykinin (Fig 9, *panels 6* and *7*) [16,18]. Bradykinin is a potent vasodilator and inducer of vascular permeability [60,61], and contact activation-induced hypotension may be an important contributor to mortality in sepsis. Indeed, Pixley *et al*. noted that administration of an anti-FXII antibody reduced hypotension and prolonged survival in a baboon model of lethal *Eschericia coli*-induced sepsis, even though it did not prevent DIC [62]. Similarly, Iwaki *et al*. reported that FXII deficiency improved hypotension but not the coagulopathy in endotoxemic mice [63]. These findings suggest that the major contribution of contact activation to morbidity and mortality in sepsis may be unrelated to thrombin generation; a conclusion consistent with our observations in the current study.

Traditionally, FXIa is considered a product of contact activation, and does not contribute directly to FXII or PK activation [15–18]. In the original cascade/waterfall models of plasma coagulation, FXI provided a unidirectional link that allows FXIIa to induce thrombin generation. The novel observations that FXI is required for maximum activation of FXII and PK during sepsis or after poly-P infusion, suggest that this interpretation may not be correct. Our *in vitro* analysis shows that FXIa can activate FXII. When considered from an evolutionary perspective, this is not surprising. FXI arose from a duplication of the PK gene [64], and the homologous FXI and PK polypeptides have similar structures [65,66]. FXI has undergone adaptations that distinguish it from PK that are important for its role in hemostasis. For example, changes to the A3 domain facilitate factor IX binding [67], while alterations in the activation site permit proteolysis by thrombin [68]. However, FXI retains important functional features of PK. Both are FXIIa substrates and circulate in complex with HK [41–43]. α-Kallikrein cleaves HK to release bradykinin [69], and FXIa also cleaves HK, although more slowly than α-kallikrein [70]. The results presented here suggest that FXII activation should be on the list of activities shared by α-kallikrein and FXIa. Griffin noted in 1978 that FXIa activates FXII on celite (diatomaceous earth), although significantly more slowly than the α-kallikrein-mediated reaction [71]. In contrast, our experiments with poly-P and nucleic acids indicate FXIa and α-kallikrein are comparable activators of FXII. The nature of the cofactor “surface” likely influences results.

The findings presented here suggest that FXI can function as a bidirectional interface between thrombin generation and contact activation. If this is the case, tissue factor-initiated thrombin generation could induce FXII activation by generating FXIa. There is convincing evidence for extensive crosstalk between coagulation and inflammation during sepsis [3,5–8], and the complex interplay between these processes provides a number of potential targets for therapeutic intervention [72,73]. Strategies directed at blocking cytokine and inflammatory responses are under active investigation, but have met with limited success in clinical trials [72,74]. Similarly, inhibiting thrombin generation with anticoagulants such as heparin may be of some benefit in subsets of patients with severe sepsis [73], but do not improve outcome in general, partly because therapy increases bleeding in patients who are already coagulopathic. FXIa is positioned at a junction between the thrombin generation and contact activation mechanisms, and its inhibition could have beneficial effects on multiple pathways that promote sepsis. Given the relatively mild bleeding disorder associated with congenital FXI deficiency in humans [15], inhibition of FXIa would not be expected to compromise hemostasis significantly and, therefore, may be safer than currently available anticoagulants in septic patients.

## Supporting Information

S1 Fig Plasma cytokine levels after CLP in WT mice. Plasma levels of **(A)** IL-6 (○) and IL-10 (●) and **(B)** IL-1β (○) and TNFα (●) at various time points after CLP in WT mice. Plasma levels of **(C)** IL-6 (black bars) and IL-10 (white bars) and **(D)** TNFα (black bars) and IL-1β (white bars) in WT mice 4 hours after CLP or sham surgery. Note that the IL-6 levels at 4 hr in CLP mice exceeded the upper limit of the assay (20 ng/ml). Error bars represent SEM. N = 3–4 mice per group.
(TIF)

S2 Fig No histologic evidence for a consumptive coagulopathy 24 hours post CLP. Photomicrographs (400X magnification) of paraffin embedded sections of mouse liver, kidney and brain stained with hematoxylin and eosin before, and 24 hours after, CLP in WT and FXI^-/-^ mice. There was no evidence of disseminated intravascular coagulation in animals of either genotype following cecal ligation and puncture.
(TIF)

S3 Fig Anti-kallikrein antibody H03. **(A)** α-Kallikrein (5 nM), FXIa (5 nM) or FXIIa (100 nM) were incubated at room temperature for 3 min with 10-fold molar excess of anti-kallikrein antibody (H03) or control vehicle (C) in a buffer with 10 μM ZnCl2, and residual activity was determined by chromogenic substrate assay. Error bars are +/− one standard deviation. **(B)** α-Kallikrein (5 nM) and 10 μM ZnCl2 were incubated with the chromogenic substrate S-2302 (200 μM) in the presence of vehicle (△), 100 nM H03 (○) or 100 nM H03 and 10 μg/ml DNA (□) and changes in OD 405 nm were monitored. **(C)** FXII (200 nM) and 10 μM ZnCl2 were incubated with vehicle (○), 5 nM α-kallikrein (➂), 5 nM α-kallikrein and 100 nM H03 (△), or 5 nM α-kallikrein with 100 nM H03 and 10 μg/ml DNA (▲) in the presence of S-2302 (200 uM). Changes in OD 405 nm were monitored. **(D)** FXII (200 nM) and 10 μM ZnCl2 were incubated with vehicle (△), 5 nM FXI (○), 5 nM FXI and 10 μg/ml DNA (□), or 5 nM FXI and 10 μg/ml DNA with 100 nM H03 (➄) in the presence of S-2302 (200 uM). Changes in OD 405 nm were monitored.
(TIF)
