# Supplementary figures and images for: Factor XI Deficiency Alters the Cytokine Response and Activation of Contact Proteases during Polymicrobial Sepsis in Mice
Source: PLoS One. 2016 Apr 5;11(4):e0152968. doi: 10.1371/journal.pone.0152968 (PMC4821616; doi:10.1371/journal.pone.0152968)

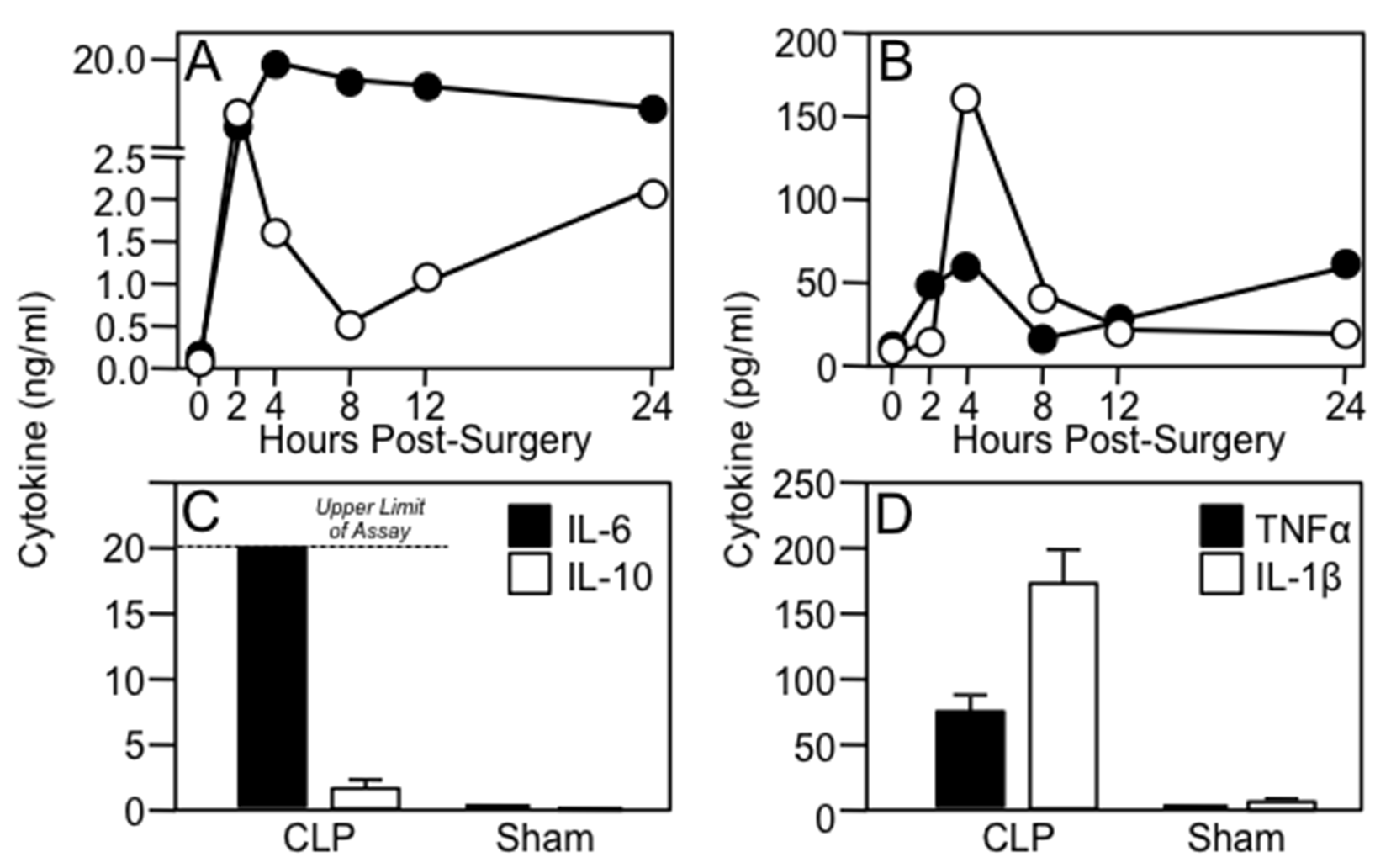

Supplement: S1 Fig — Plasma levels of (A) IL-6 (○) and IL-10 (●) and (B) IL-1β (○) and TNFα (●) at various time points after CLP in WT mice. Plasma levels of (C) IL-6 (black bars) and IL-10 (white bars) and (D) TNFα (black bars) and IL-1β (white bars) in WT mice 4 hours after CLP or sham surgery. Note that the IL-6 levels at 4 hr in CLP mice exceeded the upper limit of the assay (20 ng/ml). Error bars represent SEM. N = 3–4 mice per group. (TIF) [file pone.0152968.s001.tif]

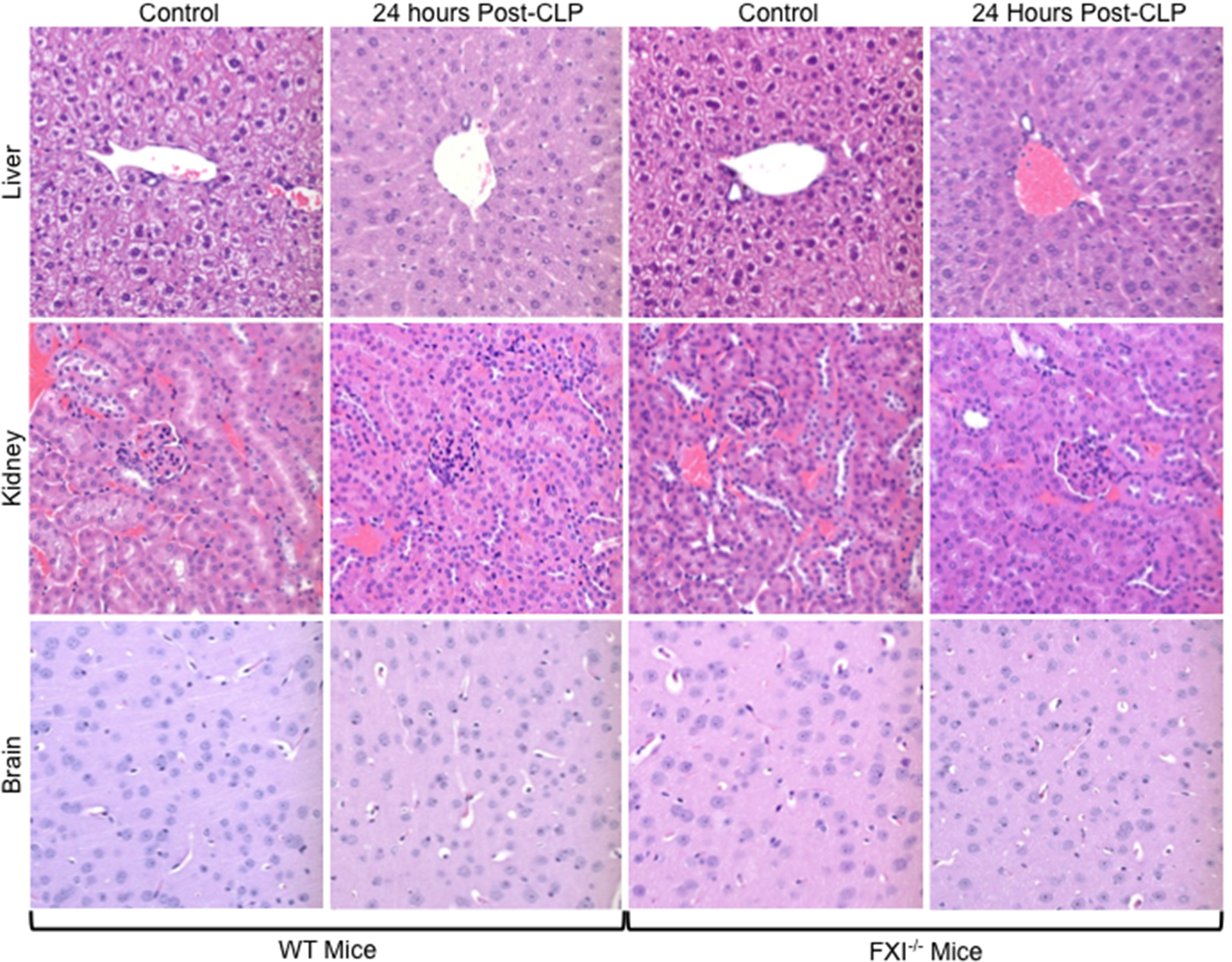

Supplement: S2 Fig — Photomicrographs (400X magnification) of paraffin embedded sections of mouse liver, kidney and brain stained with hematoxylin and eosin before, and 24 hours after, CLP in WT and FXI-/- mice. There was no evidence of disseminated intravascular coagulation in animals of either genotype following cecal ligation and puncture. (TIF) [file pone.0152968.s002.tif]

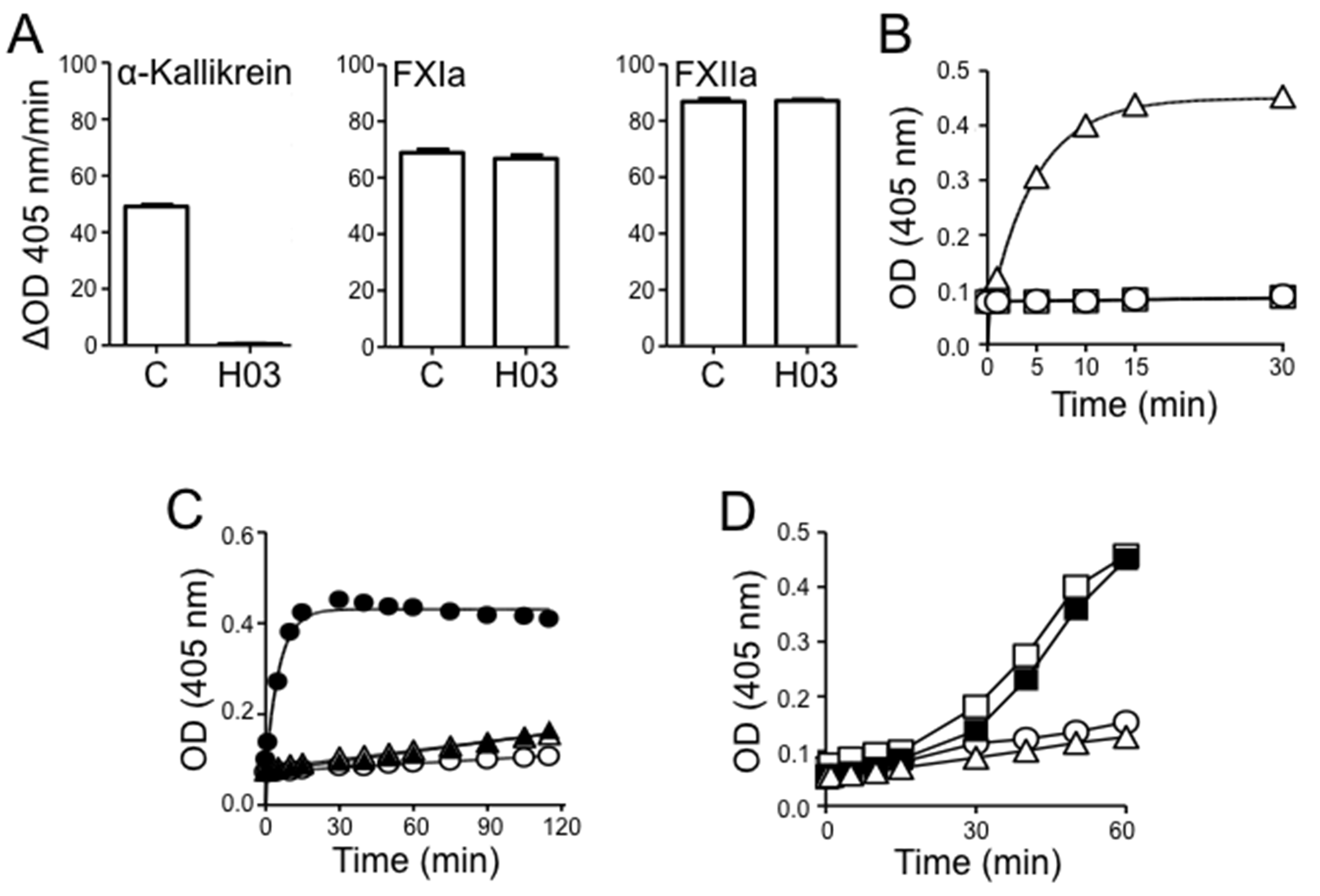

Supplement: S3 Fig — (A) α-Kallikrein (5 nM), FXIa (5 nM) or FXIIa (100 nM) were incubated at room temperature for 3 min with 10-fold molar excess of anti-kallikrein antibody (H03) or control vehicle (C) in a buffer with 10 μM ZnCl2, and residual activity was determined by chromogenic substrate assay. Error bars are +/− one standard deviation. (B) α-Kallikrein (5 nM) and 10 μM ZnCl2 were incubated with the chromogenic substrate S-2302 (200 μM) in the presence of vehicle (△), 100 nM H03 (○) or 100 nM H03 and 10 μg/ml DNA (□) and changes in OD 405 nm were monitored. (C) FXII (200 nM) and 10 μM ZnCl2 were incubated with vehicle (○), 5 nM α-kallikrein (➂), 5 nM α-kallikrein and 100 nM H03 (△), or 5 nM α-kallikrein with 100 nM H03 and 10 μg/ml DNA (▲) in the presence of S-2302 (200 uM). Changes in OD 405 nm were monitored. (D) FXII (200 nM) and 10 μM ZnCl2 were incubated with vehicle (△), 5 nM FXI (○), 5 nM FXI and 10 μg/ml DNA (□), or 5 nM FXI and 10 μg/ml DNA with 100 nM H03 (➄) in the presence of S-2302 (200 uM). Changes in OD 405 nm were monitored. (TIF) [file pone.0152968.s003.tif]
